# Increasing the Availability of Psychological Treatments: A Multinational Study of a Scalable Method for Training Therapists

**DOI:** 10.2196/10386

**Published:** 2018-06-08

**Authors:** Marianne O'Connor, Katy E Morgan, Suzanne Bailey-Straebler, Christopher G Fairburn, Zafra Cooper

**Affiliations:** ^1^ Centre for Research on Dissemination at Oxford Department of Psychiatry University of Oxford Oxford United Kingdom; ^2^ Department of Medical Statistics London School of Hygiene and Tropical Medicine University of London London United Kingdom; ^3^ Department of Psychiatry Yale School of Medicine Yale University New Haven, CT United States

**Keywords:** internet, web-centered, eating disorders, cognitive therapy, effective treatment

## Abstract

**Background:**

One of the major barriers to the dissemination and implementation of psychological treatments is the scarcity of suitably trained therapists. A highly scalable form of Web-centered therapist training, undertaken without external support, has recently been shown to have promise in promoting therapist competence.

**Objective:**

The aim of this study was to conduct an evaluation of the acceptability and effectiveness of a scalable independent form of Web-centered training in a multinational sample of therapists and investigate the characteristics of those most likely to benefit.

**Methods:**

A cohort of eligible therapists was recruited internationally and offered access to Web-centered training in enhanced cognitive behavioral therapy, a multicomponent, evidence-based, psychological treatment for any form of eating disorder. No external support was provided during training. Therapist competence was assessed using a validated competence measure before training and after 20 weeks.

**Results:**

A total of 806 therapists from 33 different countries expressed interest in the study, and 765 (94.9%) completed a pretraining assessment. The median number of training modules completed was 15 out of a possible 18 (interquartile range, IQR: 4-18), and 87.9% (531/604) reported that they treated at least one patient during training as recommended. Median pretraining competence score was 7 (IQR: 5-10, range: 0-19; N=765), and following training, it was 12 (IQR: 9-15, range: 0-20; N=577). The expected change in competence scores from pretraining to posttraining was 3.5 (95% CI 3.1-3.8; *P*<.001). After training, 52% (300/574) of therapists with complete competence data met or exceeded the competence threshold, and 45% (95% CI 41-50) of those who had not met this threshold before training did so after training. Compliance with training predicted both an increase in competence scores and meeting or exceeding the competence threshold. Expected change in competence score increased for each extra training module completed (0.19, 95% CI 0.13-0.25), and those who treated a suitable patient during training had an expected change in competence score 1.2 (95% CI 0.4-2.1) points higher than those who did not. Similarly, there was an association between meeting the competence threshold after training and the number of modules completed (odds ratio, OR=1.11, 95% CI 1.07-1.15), and treating at least one patient during training was associated with competence after training (OR=2.2, 95% CI 1.2-4.1).

**Conclusions:**

Independent Web-centered training can successfully train large numbers of therapists dispersed across a wide geographical area. This finding is of importance because the availability of a highly scalable method of training potentially increases the number of people who might receive effective psychological treatments.

## Introduction

In recent years, there has been considerable progress in developing evidence based psychological treatments. This progress has highlighted two important issues: the need to disseminate these treatments developed in controlled settings to routine clinical care [1,2] and the need to ensure that more people in need of care actually receive effective treatment [3].

Psychological treatments are difficult to disseminate [3,4] and implement widely*.* One of the major barriers to their dissemination and implementation is the scarcity of suitably trained therapists [5]*.* The currently accepted method of training typically involves attending a specialist workshop, reading relevant texts, and a subsequent period of supervision from someone expert in the treatment [6]. As this method is both labor-intensive and costly [7,8] and vulnerable to the shortage of treatment experts, it limits the number of therapists that can be trained, and therefore, the number of people who might potentially receive effective treatment.

There has been growing interest in addressing the problem of the scalability of training by using the internet to train therapists [9,10]. A recent systematic review has provided preliminary support for Web- based training methods while noting significant methodological shortcomings in many studies [11], particularly the use of outcome measures developed specifically for a particular study without the assessment and reporting of their psychometric properties [12].

Web-based training methods have a number of potential advantages. Training can be offered simultaneously to large numbers of geographically dispersed trainees with training materials that can be accessed at any time and from any place. Trainees can review and revisit material in a way that potentially reinforces learning and prevents subsequent therapist drift [13,14]. In addition, trainees can view detailed clinical illustrations of treatment interventions and complete interactive formative assessments such as knowledge tests that facilitate experiential learning. Furthermore, the training program can be updated regularly to incorporate new information. Finally, the training process can be informed by and improved upon by the collection of data on website usage.

We have developed and tested a specific form of Web-centered therapist training [15,16]. It differs from conventional training in that the training is fully automated with the expertise residing within the program rather than being provided by an outside expert. Web-centered training can be undertaken completely autonomously (independent training) or with support from a nonspecialist support worker whose role is to encourage the trainee to follow the program. An initial proof-of-concept study of the supported form of Web-centered training found that the method was feasible and acceptable to trainees across Ireland and was effective in promoting therapist competence, with 43% of trainee therapists scoring above the threshold of a validated measure of therapist competence after training [16]. This finding was replicated and extended in a randomized controlled comparison that recruited therapists across the United States and Canada and compared the independent and supported form of training [17]. Both methods of training were effective in improving therapist competence, with 45% of therapists becoming competent after training and no clear evidence of a change in competence scores between the end of training and 6-month follow-up.

This study had two aims: (1) to determine whether our previous finding concerning acceptability and efficacy of the most highly scalable form of training (independent training) could be replicated in a multinational sample of therapists who wished to be trained and (2) to investigate the characteristics of the therapists who were most likely to benefit from this form of training.

## Methods

### Design

A cohort of eligible therapists was offered access to the 20-week, Web-centered training program. No external support was provided during the training. Therapist competence was assessed before and after the training.

The research protocol was submitted to Oxford University Central Research Ethics Committee. As the intervention was judged to be educational rather than clinical, the committee decided that formal ethical approval was not required.

### Recruitment

Participants were recruited internationally through a posting on the research group’s website offering free training in enhanced cognitive behavioral therapy (CBT-E), a multicomponent evidence-based psychological treatment for any form of eating disorder [18,19]. The posting included a link to an online description of the training and the research study. Potential participants had to be licensed mental health professionals who were prepared to take part in the research evaluating this training and who provided informed digital consent.

It was strongly recommended that participants met the following eligibility criteria: had been previously trained in delivering a short-term psychological treatment, were currently working with people with eating disorders, were willing to devote at least 9 hours to the training program, and were able to treat one or more suitable patients using CBT-E during the 20-week period of training. In the information provided to participants, it was stressed that clinical responsibility for their patients remained with their local clinical team and not with the researchers.

Participants were asked to complete a brief online questionnaire asking about their professional background, age, gender, and clinical experience. They also completed an online therapist competence assessment measure (see below). They were subsequently sent a link to the training website together with instructions about how to use the training program. In addition, they were sent brief information about the minimum technical specifications for accessing and using the website.

Recruitment took place over 18 months from August 2016 to January 2017.

### The Training Program

The CBT-E Web-centered training program has two main parts: The Course and The Library. Fuller details are provided elsewhere [16], and a summary of the content of the training is provided in Multimedia Appendix 1. Briefly, the Course is linear in nature and takes between 8 and 9 hours to complete. It is a detailed practical description of how to implement the main focused form of CBT-E given by an expert on the treatment (CGF). This description is delivered in the form of multiple brief video presentations accompanied by handouts and interspersed with formative learning exercises, video recordings of acted illustrations of the treatment, and tests of knowledge together with feedback. While working through The Course, trainees are encouraged to read relevant sections from the treatment manual [20] and treat one or more patients.

The second part of the training website, The Library, contains all the material in The Course, including the handouts, learning exercises, and clinical illustrations in indexed form. In addition, there is a large amount of supplementary material on how to use CBT-E with specific subgroups of patients including adolescents; those who are severely underweight; and those with clinical perfectionism, core low self-esteem, or marked interpersonal difficulties. Participants were granted access to The Course and core Library material from the start of training. They only had access to the supplementary Library material once they had completed the study.

Trainees were not provided with any external support while completing the training. They received reminder emails 6, 10, 14, and 18 weeks after starting, informing them of the number of weeks of training that had elapsed and the number of weeks remaining.

### Assessment

Participants’ competence at delivering CBT-E, ie, their knowledge of how to deliver the treatment skilfully [7], was assessed before training and immediately following training. It was measured using a scalable online measure with sound psychometric properties that had previously been developed independently of the creation of the training website. Its development and validation are described in detail in a separate publication [21]. This included detailed blueprinting, state-of-the-art item writing, independent item review, and initial field-testing, followed by formal Rasch analysis to test for good model fit. Strict criteria of unidimensionality were met by stepwise exclusion of misfitting items until there was no individual item misfitting at *P*<.01. The resulting measure consists of 22 items addressing trainees’ knowledge and understanding of CBT-E and its implementation (ie, applied knowledge). The instrument generates a total score (out of a possible 22), and trainees can be classified as scoring at or above the previously established cut point. This was established using receiver operator characteristic analyses to determine the “best cut point” from the values of sensitivity and specificity calculated at increasing test score cut points. Three equivalent versions of the competence measure were developed so that different versions could be used on different assessment occasions as was done in this study. Those who score above the competence threshold (an equated score of 12 or above) have also been shown to be competent at implementing CBT-E when systematically observed and rated using a performance based measure [22].

### Data Analysis

Missing values were imputed using multiple imputation (see Multimedia Appendix 2). Those with a nonmissing pretraining score, gender, and age were used for all analyses based on imputed data (N=760). Fifty imputations were created, and regression models were applied to each imputed dataset, and the estimates from each imputation were combined using Rubin’s rules [23]. Imputed values for posttraining score were derived from the imputed values for change in score.

Linear regression models were used to investigate changes in score after training and logistic regression models to explore scoring above the competence cut point after training.

To examine predictors of change in score and competence levels after training, linear and logistic models were fitted adjusting for age and gender and, additionally, in each case, a second set of models were fitted adjusting for pretraining score. The variables examined, included in the models one at a time, were as follows: trainee characteristics (professional background, years of clinical experience, weekly time spent treating patients having attended a training workshop, and using English to treat patients) and training adherence (number of training modules completed, treating at least one suitable patient during training, and number of patients treated during training). The number of modules completed and treating a suitable patient were also examined together to explore the associations for each of these variables when adjusting for the other. Reported results use imputed data unless otherwise stated. Preliminary analyses explored the impact of country of recruitment on change in score and, as no effect was found, it was not included in further analyses. After exploring various distributional forms to describe the number of training modules completed, it was decided to use this variable as a linear term.

To explore attrition, univariate logistic regression models were fitted to examine associations between a binary variable for having a missing posttraining test score and a range of trainee adherence variables and trainee characteristics as detailed above.

All analyses were conducted in Stata 15 (StataCorp LLC), with multiple imputation performed using the smcfcs command [24].

## Results

### Recruitment

**Figure 1:**
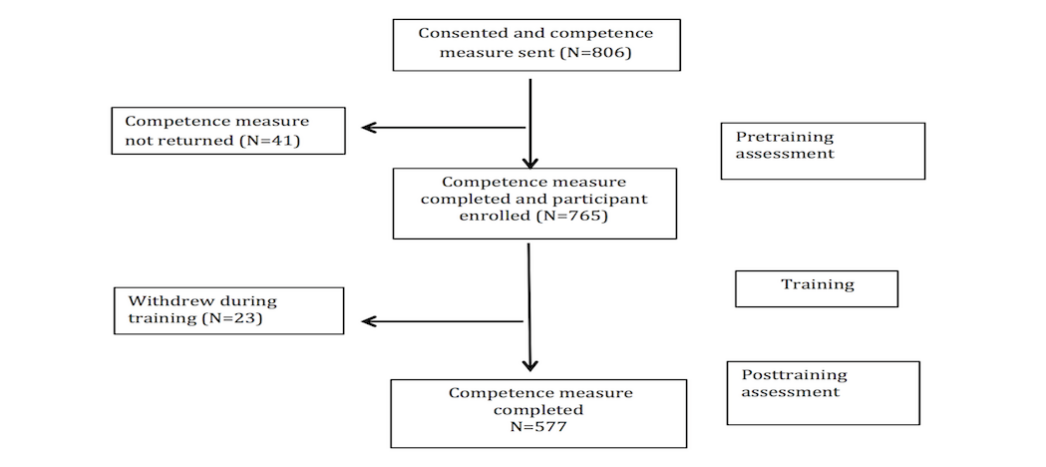
Flow of the participants through the study.

A total of 806 therapists expressed an interest in participating in the study and completed the consent form. Of these 765 (94.9%, 765/806) completed the pretraining assessment and were given access to the training website. Figure 1 shows their progress through the study.

The median age of the participants was 36 years (interquartile range, IQR: 31-44; range: 22-67), and 88.7% were female (691/779). As regards professional training, those with a clinical psychology background were the overwhelming majority at 47.7% (373/782), followed by psychiatric nurses comprising 8.6% (67/782) and those from a social work background at 7.8% (61/782). The remainder came from a variety of other backgrounds including counselling, CBT therapist training, family therapy, psychiatry, nursing, and dietetics. Further details about their professional background, clinical experience, current clinical practice, and training can be seen in Table 1.

**Table 1:** Background characteristics of trainee therapists.

Characteristics		Statistics
**Gender (N=779), n (%)**		
	Female	691 (88.7)
	Male	88 (11.3)
Age in years (N=780), median (IQR^a^; range)		36 (31-44; 22-67)
**Professional background (N=782), n (%)**		
	Clinical psychologist PhD	99 (12.7)
	Clinical psychologist PsyD	165 (21.1)
	Clinical psychologist Masters	109 (13.9)
	Psychiatric nurse	67 (8.6)
	Social worker	61 (7.8)
	Other	281 (35.9)
Hours per week seeing patients (N=729), median (IQR; range)		10 (4-17; 0-40)
Years of clinical experience (N=718), median (IQR; range)		6 (3-10; 0-45)
**Previously attended a CBT-E^b^ workshop (N=778), n (%)**		
	Yes	212 (27.3)
	No	566 (72.8)
**Primary language for treating patients (N=766), n (%)**		
	English	580 (75.7)
	Other	186 (24.3)

^a^IQR: interquartile range.

^b^CBT-E: enhanced cognitive behavioral therapy.

The trainee therapists were located in 33 different countries. The majority, 76.6% (617/806), came from English-speaking countries (United Kingdom, United States, Australia, Canada, New Zealand, and Ireland, respectively), followed by 5.8% (47/806) from the Netherlands, 4.1% (33/806) from Sweden, 3.7% (30/806) from Italy, 2.6%(21/806) from Norway, and 7.2% (58/806) from other countries.

### Training Completion

The median number of modules of the training program completed was 15 out of a possible 18 (IQR: 4 -18), and 87.9% (531/604) of the trainees reported that they treated at least one suitable patient during the training with 42.9 % (259/604) reporting that they treated two or more.

### Outcome of Training

The median pretraining competence score was 7 (IQR: 5-10, range: 0-19; N=765), and following training, it was 12 (IQR: 9-15, range: 0-20; N=577). The expected change in competence scores from pretraining to posttraining was 3.5 (95% CI 3.1-3.8; *P*<.001).

After training, 52% (300/574) of therapists with complete competence data met or exceeded the competence threshold. Of those with complete data, 45% (213/471, 95% CI 41-50%) who did not meet the competence threshold before training did so after training, and 16% (16/103, 95% CI 9-24%) who did meet it before training no longer met it after training.

### Predictors of Training Outcome

#### Change in Competence Score

As can be seen in Table 2 age, gender, years of clinical experience, time spent treating patients each week, and professional background were not associated with changes in expected competence scores whether adjusting for pretraining score or not.

When adjusting for pretraining scores, neither prior workshop attendance nor using English to treat patients was associated with expected change in competence scores. When unadjusted for pretraining scores prior workshop attendance was associated with a reduction of 1.6 points in expected change (95% CI 0.9-2.3, *P*<.001) and using English to treat patients was associated with a 0.9 increase in expected change (95% CI 0.2-1.6, *P*=.02), suggesting that these differences were primarily due to an association with pretraining score. Similarly, when adjusting for pretraining score, the association between number of patients treated while training was not statistically significant (*P*=.16), although when unadjusted, there was borderline evidence supporting this association with change in score (*P*=.03).

There was strong evidence of an increase in expected change in competence score for each extra training module completed, whether or not adjustments were made for pretraining score (0.14 [95% CI 0.07-0.20] and 0.19 [95% CI 0.13-0.25] points per module, respectively). Treating a suitable patient while training was associated with change in competence score when adjusting for pretraining score with those who treated such a patient having a change in score 1.2 (95% CI 0.4-2.1) points higher than those who did not. The effect sizes did not appear to change when the number of modules completed and treating a suitable patient were examined together (0.18 [95% CI 0.13-0.23, *P*<.001] and 1.3 [95% CI 0.5-2.2, *P*=.001], respectively; N=601 therapists with nonmissing data).

#### Scoring Above the Competence Threshold

An examination of the associations between trainee characteristics and training adherence variables and scoring above the competence threshold yielded a pattern of results similar to those for the predictors of expected change in score.

Age, gender, years of clinical experience, and time spent treating patients each week were not associated with competence after training, whether adjusting for pretraining score or not (see Table 3).

When adjusting for pretraining score, there was no evidence for an association between professional background and competence (*P*=.22). When unadjusted for pretraining score, professional background was associated with competence after training (*P*<.001), suggesting that this may be due to differences in pretraining score by professional background. Similarly, there was no evidence of an association between prior workshop attendance or using English to treat patients and posttraining competence when adjusting for pretraining score. When unadjusted, attending a workshop increased the odds of achieving competence after training by 3.2 (95% CI 2.2-4.6), and using English decreased the odds by 0.5 (95% CI 0.4-0.8).

Again, as for the predictors of change in competence score, there was strong evidence of an association between being above the competence threshold after training and the number of modules completed with the odds ratio (OR) when adjusting for pretraining scores being very similar to that when unadjusted for pretraining score (OR=1.11, 95% CI 1.07-1.15 and OR=1.11, 95% CI 1.08-1.15, respectively; see Table 3). When adjusting for pretraining score, there was evidence that treating at least one suitable patient while training was associated with competence after training (OR=2.2, 95% CI 1.2-4.1) and only borderline evidence for an association with number of patients treated (*P*=.05).

**Table 2:** Predictors of change in competence score after training.

Trainee characteristic or training compliance			Unadjusted for pretraining score		Adjusted for pretraining score	
			Difference in expected change score (95% CI)	*P* value	Difference in expected change score (95% CI)	*P* value
Age (years)			0.00 (−0.03 to 0.03)	.98	−0.01 (−0.04 to 0.02)	.52
**Gender**						
	Female		—	—	—	—
	Male		0.1 (−0.9 to 1.1)	.87	0.1 (−0.9 to 1.0)	.91
Clinical experience (years)			0.04 (−0.02 to 0.09)	.24	0.04 (−0.01 to 0.10)	.11
Weekly time treating patients (hours)			−0.03 (−0.06 to 0.01)	.10	−0.01 (−0.04 to 0.02)	.54
Professional background^a^						
	**Clinical psychology**					
		PsyD	—	—	—	—
		PhD	1.3 (0.2-2.4)^b^	.02	0.7 (−0.2 to 1.7)^b^	.14
		Masters	0.3 (−0.8 to 1.3)^b^	.64	−0.3 (−1.3 to 0.6)^b^	.51
	Psych nurse		0.3 (−1.0 to 1.6)^b^	.62	−0.9 (−2.1 to 0.2)^b^	.11
	Social work		0.4 (−0.9 to 1.7)^b^	.56	−0.9 (−2.1 to 0.3)^b^	.14
	Other		0.5 (−0.3 to 1.4)^b^	0.21	−0.4 (−1.2 to 0.4)^b^	.30
Treating patients using English			0.9 (0.2-1.6)	.02	−0.1 (−0.8 to 0.5)	.72
Previous attendance at workshop			−1.6 (−2.3 to −0.9)	<.001	0.2 (−0.5 to 0.9)	.54
**Number of patients treated during training (n=572)^c,d^**						
	No patients		—	—	—	—
	One patient		−0.1 (−1.4 to 1.3)^e^	.94	0.7 (−0.4 to 1.9)^e^	.21
	Two or more patients		−0.5 (−1.8 to 0.7)^e^	.40	0.9 (−0.2 to 2.0)^e^	.10
	One or more part cases		0.6 (−0.7 to 1.9)^e^	.36	1.3 (0.1-2.4)^e^	.03
Number of training modules completed^f^			0.14 (0.07-0.20)	<.001	0.19 (0.13-0.25)	<.001
Treating at least one suitable patient (n=572)^c^			0.4 (−0.6 to 1.4)	.43	1.2 (0.4-2.1)	.004

^a^Overall *P* value for including professional background when unadjusted for pretraining score, *P*=.34 and when adjusted for pretraining score, *P*=.06.

^b^Effect relative to clinical psychology (PsyD).

^c^Analyses conducted on complete cases only.

^d^Overall *P* value for including number of patients; *P*=.03 when unadjusted for pretraining score and *P*=.16 when adjusted.

^e^Effect relative to treating no patients.

^f^Number of modules completed included as a linear term.

### Predictors of Training and Study Dropout

About a quarter of therapists who completed initial study assessments (186/760, 24.5%) did not complete competence assessments after training. Not surprisingly, the number of training modules completed was strongly associated with the odds of having a missing posttraining assessment, decreasing by over 25% (OR=0.73, 95% CI 0.70-0.76; *P*<.001) for every extra module viewed. Pretraining competence score was also strongly associated with having a missing posttraining score, with the odds decreasing by around 10% for every extra point scored at pretraining (OR=0.89, 95% CI 0.85-0.94, *P*<.001). When adjusting the model to take account of the number of modules completed, the OR for the number of training modules completed remains almost identical (OR=0.73, 95% CI 0.70-0.77; *P*<.001), whereas the OR for pretraining score is greatly attenuated (OR=0.94, 95% CI 0.88-1.00; *P*=.07), suggesting that the decrease in missing posttraining scores for those with higher pretraining scores may be partially mediated through the number of modules completed. For further details of trainee background characteristics associated with missing posttraining scores, see Multimedia Appendix 3.

**Table 3:** Predictors of competence after training.

Trainee characteristic or training compliance			Unadjusted for pretraining score		Adjusted for pretraining score	
			OR^a^ (95% CI)	*P* value	OR (95% CI)	*P* value
Age (years)			0.99 (0.97-1.01)	.23	0.99 (0.98-1.01)	.57
**Gender**						
	Female		—	—	—	—
	Male		0.96 (0.59-1.58)	.89	0.94 (0.53-1.67)	.84
Clinical experience (years)			1.03 (1.00-1.06)	.10	1.03 (0.99-1.06)	.12
Weekly time treating patients (hours)			1.01 (1.00-1.03)	.13	1.00 (0.98-1.02)	.80
**Professional background^b^**						
	**Clinical psychology**					
		PsyD	—	—	—	—
		PhD	0.8 (0.4-1.3)^c^	.37	1.0 (0.5-1.9)^c^	.92
		Masters	0.5 (0.3-0.9)^c^	.03	0.7 (0.3-1.2)^c^	.18
	Psych nurse		0.3 (0.1-0.6)^c^	<.001	0.5 (0.2-1.1)^c^	.07
	Social worker		0.3 (0.2-0.6)^c^	.001	0.5 (0.2-1.1)^c^	.09
	Other		0.5 (0.3-0.8)^c^	.002	0.7 (0.5-1.2)^c^	.25
Treating patients using English			0.5 (0.4-0.8)	.001	0.9 (0.6-1.4)	.72
Previous attendance at workshop			3.2 (2.2-4.6)	<.001	1.3 (0.8-2.0)	.25
**Number of patients treated during training (n=572)^d,e^**						
	No patients		—	—	—	—
	One patient		1.8 (0.8-3.9)^f^	.13	1.2 (0.5-2.7)^f^	.67
	Two or more patients		3.5 (1.7-7.2)^f^	.001	1.9 (0.9-4.1)^f^	.11
	One or more part cases		2.7 (1.3-5.7)^f^	.009	2.2 (1.0-4.8)^f^	.06
Number of training modules completed^g^			1.11 (1.08-1.15)	<.001	1.11 (1.07-1.15)	<.001
Treating at least one suitable patient (n=572)^d^			3.0 (1.7-5.2)	<.001	2.2 (1.2-4.1)	.009

^a^OR: odds ratio.

^b^Overall value for professional background when unadjusted for pretraining score: *P*<.001 and when adjusted for pretraining score: *P*=.22.

^c^Effect relative to clinical psychology (PsyD).

^d^Analyses conducted on complete cases only.

^e^Overall value for number of patients seen when unadjusted for pretraining score: *P*<.001 and when adjusted for pretraining score: *P*=.05.

^f^Effect relative to treating no patients.

^g^Number of modules completed included as a linear term.

## Discussion

### Principal Findings

The study had two aims. The first was to determine whether earlier findings concerning the acceptability and effectiveness of a highly scalable form of Web-centered training (independent training) could be replicated in a multinational sample of therapists. This was found to be the case with the present findings replicating the earlier ones. Over 70% of the therapists enrolled in the study provided end of training competence data, the great majority of the training modules were completed, and most therapists treated a suitable patient while training in compliance with the recommendations of the program. Scores on the validated competence measure increased significantly, and 45% of the trainee therapists who had not previously been competent achieved competence scores indicative of a good level of competence. This figure is identical to that obtained in the study of therapists in the United States and Canada [17].

The second aim was to investigate the characteristics of the therapists who were most likely to benefit from this form of training. Compliance with training recommendations was a predictor of both an increase in competence score and becoming competent. There was a linear relationship between the number of training modules completed and changes in competence score, and the likelihood of therapists scoring above the competence threshold increased with the number of modules they completed. Similarly, treating at least one patient while training was associated with both an increased change in competence score and the likelihood of scoring over the competence threshold.

It was less clear from these findings why some therapists complied with the training recommendations, thereby deriving benefit, while others did not. An examination of those who were study noncompleters seems to suggest that any efforts to improve training should focus on ways of increasing training completion in those groups at risk of not doing so. It is possible that for these groups, the addition of a more generic introduction to the treatment method to complement the focus on specific specialist skills related to providing evidence-based CBT-E might be beneficial. Such an addition might also allow the training to be extended to an even wider range of potential therapists.

### Comparisons With Other Studies

There has been limited research on the outcome of therapist training against which to compare the present findings [8,13,25]. In general, therapist training has been relatively neglected as a research topic until recently [2,26], and Web-based training is an even more novel focus of interest [11].

Competence figures that have been reported following more conventional training in psychological treatment for depression range from 21% after attending a training workshop to 96% after extensive consultation with an expert including treatment session review and feedback [27]. A study of community clinicians receiving training in transdiagnostic cognitive behaviour therapy reported 59.5% of clinicians competent after training [28]. However, the latter training also involved extensive expert consultation and session review of a kind that is not scalable; thus, the findings are not directly comparable. Although positive changes in knowledge and skill have been reported for Web-based training, studies have employed widely varying methods, and few have employed standardized outcome measures, making it difficult to draw firm conclusions [11,12]. One study of Web-based training in transdiagnostic cognitive behavior therapy for community clinicians that used an established measure reported that 48% reached competency, but the training also included 7.5 months of face-to-face consultation with previously trained peers and 6.5 hours of expert instructor time [29]. Clearly, these findings are not directly comparable with the independent training investigated in this study.

### Study Strengths

The study had a number of strengths. First, a large number of trainees from 33 countries were recruited and trained. Second, nearly three-quarters of those who consented to participate in the study completed competence assessments at the end of training. Third, the study used a previously validated measure of therapist competence that had an empirically established competence threshold. Fourth, the study was able to explore predictors of change in competence scores and the achievement of competence, as well as factors associated with the noncompletion of the study and training.

### Study Limitations

The study also had certain limitations. First, it did not include a no-training control condition or a delayed training group. Thus, we cannot discount the possibility that competence scores would have increased over time without any training, but this seems unlikely especially given the strong association between module completion and competence scores. Second, despite good retention in the study, there was still a significant amount of missing data, necessitating the use of multiple imputation that relies on certain assumptions about the nature of the missing data and, in some cases, the use of analyses based on complete data only. Attrition, both in the form of participants ceasing to use an intervention and not completing study assessments, has been noted as a particular problem in internet interventions [30]. Third, as clinicians chose to undertake the training, generalization to those less motivated may not be warranted. Fourth, although we have reported competence data concerning clinicians’ ability to provide CBT-E treatment, we do not have data concerning the quality of the treatment they actually provide.

### Conclusions

This study confirms that independent Web-centered training can successfully train a large number of therapists dispersed across a wide geographical area. This finding is of great practical importance because it indicates that this form of independent Web-centered training is able to overcome a major barrier to the dissemination and implementation of psychological treatments. The availability of a highly scalable training method potentially greatly increases the number of people who might have access to effective psychological treatments.

## Appendix

Multimedia Appendix 1 Enhanced cognitive behavioral therapy (CBT-E) training program.

Multimedia Appendix 2 Data imputation.

Multimedia Appendix 3 Predictors of training and study dropout.

## Abbreviations

CBT-E: enhanced cognitive behavioral therapy
IQR: interquartile range
OR: odds ratio
