# Supplementary material for: Increasing the Availability of Psychological Treatments: A Multinational Study of a Scalable Method for Training Therapists
Source: J Med Internet Res. 2018 Jun 8;20(6):e10386. doi: 10.2196/10386 (PMC6015265; doi:10.2196/10386)
Supplement: Multimedia Appendix 2 [file jmir_v20i6e10386_app2.pdf]

Multimedia Appendix 2- Data imputation

Multiple imputation was used to create 50 imputed data sets for the 760 therapists with a non-missing pre-training score, gender and age, using the Stata package smcfcs [1]. The imputation model used change in score from pre- to post-training as the outcome variable, and included pre-training score, number of training modules completed (as linear, square and cubic terms), country of recruitment, age, gender, professional background, weekly time spent treating patients, years of clinical experience, previously attending a workshop, and primarily using English to treat patients as covariates. Covariates included in the imputation model with missing data were also imputed (weekly time spent treating patients, years of clinical experience, previously attending a workshop, and primarily using English with patients). Country of recruitment was not used as a predictor variable when imputing using English to treat patients since these two variables were highly collinear, leading to convergence problems for the imputation model. Two other categorical variables (seeing an eligible patient during training and number of patients seen during training) were also highly collinear, and so were not included in the imputation model. Analyses for these training variables were therefore carried out on complete cases only.

Imputed values for post-training score were calculated from pre-training score and the imputed values of change. Imputed values for post-training competence were calculated based on the imputed post-training score, and the cut-points for competence.

1. Bartlett JW, Morris TP. Multiple imputation of covariates by fully conditional specification: Accommodating the substantive model. Stata J 2015;15(2):437–456.
