# Supplementary material for: Increasing the Availability of Psychological Treatments: A Multinational Study of a Scalable Method for Training Therapists
Source: J Med Internet Res. 2018 Jun 8;20(6):e10386. doi: 10.2196/10386 (PMC6015265; doi:10.2196/10386)
Supplement: Multimedia Appendix 3 [file jmir_v20i6e10386_app3.pdf]

Multimedia Appendix 3- Predictors of training and study drop out[1]

	OR (95% CI)	P-value
Trainee characteristic		
Age in years (adjusted for gender)	1.00 (0.98 to 1.01)	0.73
Gender (adjusted for age)		
- Female		
- Male	1.55 (0.95 to 2.51)	0.08
Country of recruitment[2]		
- UK		
- US	1.46 (0.91 to 2.34)[3]	0.11
- Australia	1.56 (0.94 to 2.57)[3]	0.08
- Other	0.44 (0.29 to 0.68)[3]	<0.001
Clinical experience (years), N =699	0.97 (0.95 to 1.00)	0.08
Weekly time treating patients (hours), N=709	0.99 (0.97 to 1.01)	0.41
Professional background[4]		
- Clinical psychology - PsyD		
- PhD	0.87 (0.46 to 1.64)[5]	0.67
- Masters	1.73 (0.99 to 3.02)[5]	0.06
-Psych nurse	2.01 (1.07 to 3.79)[5]	0.03
-Social worker	2.03 (1.06 to 3.89)[5]	0.03
-Other	1.00 (0.62 to 1.62)[5]	0.99

Treating patients using English, N=745	3.0 (1.8 to 4.8)	<0.001
Previous attendance at workshop, N=757	0.61 (0.41 to 0.91)	0.02

[1]Some analyses in this section were conducted on complete cases. When the number of trainees differs from 760, the number included is noted in the table. .
[2]Overall p-value for country of recruitment: p < 0.001
[3]Effect relative to UK
[4]Overall p-value for professional background: p = 0.01
[5]Effect relative to clinical psychology (PsyD)

As can be seen in the table above a number of background characteristics were associated with having a missing post-training competence score. These were: country of recruitment, with those recruited from countries other than the UK, USA or Australia much less likely to have missing scores compared to those from the UK (OR: 0.44, 95% CI: 0.29 to 0.68 *P*<0.001); professional background, with psychiatric nurses and social workers somewhat more likely to have missing scores than clinical psychologists (PsyD) (OR 2.01 95% Cl 1.07 to 3.79 *P*=0.03 and OR 2.03 95% Cl 1.06 to 3.89 *P*=0.03 respectively); previously attending a workshop, with those who had done so less likely to have missing scores (OR: 0.61, 95% CI: 0.41 to 0.91 *P*=0.02); and using English to treat patients, with those who used English more likely to have missing post training scores (OR: 3.0, 95% CI: 1.8 to 4.8 *P*<0.001).

.
